# Incorporating ‘reason for use’ into the prescribing process of medication: a survey on the opinion of patients in Flanders, Belgium

**DOI:** 10.1186/s12913-022-08596-w

**Published:** 2022-09-30

**Authors:** Marijke Peeters, Elias Iturrospe, Dominique Jans, Alexander L. N. van Nuijs, Hans De Loof

**Affiliations:** 1 University of Antwerp, Toxicological Centre, Universiteitsplein 1, 2610 Antwerp, Belgium; 2 Department of In Vitro Toxicology and Dermato-cosmetology, Vrije Universiteit Brussel, Laarbeeklaan 103, 1090 Jette, Belgium; 3 University of Antwerp, Laboratory of Physiopharmacology, Universiteitsplein 1, 2610 Antwerp, Belgium

**Keywords:** Drug safety, Medicine policy, Pharmacy, Medication indication, Multidisciplinary

## Abstract

**Background:**

A longstanding debate exists about including a ‘reason for use’ on prescriptions for medication. Little is known, however, about patients’ opinions on this subject.

**Methods:**

An internet-based questionnaire, consisting mainly of Likert scale questions, was distributed online to the general public in Belgium. Results from 1034 responses were analyzed using descriptive statistics.

**Results:**

Opinions from patients toward including a ‘reason for use’ on medication prescriptions were generally positive. A clear majority of 62% increased to 74% after providing information about the possible link between indication and medication dose. A majority of the participants expressed a positive attitude regardless of the pathology involved, although sexually transmitted diseases were of greatest concern. Other important aspects differentiating the opinion positively was the transmission of this information in an electronic-only form and limiting it to the regular pharmacist excluding further use by third parties such as other pharmacies or insurance companies. Patients using multiple medicines and those frequenting the same pharmacy also had a more favorable opinion about including the reason for use. In addition, analysis of physician and pharmacist questionnaire responses, explicitly excluded from the main analysis, confirmed the known contrasting opinions in these subgroups.

**Conclusions:**

Patients have strong support for transferring information on the ‘reason for use’ of their prescriptions to their regular pharmacy if this is done in a secure and privacy-conscious way enabling increased patient safety and improved pharmaceutical care.

**Supplementary Information:**

The online version contains supplementary material available at 10.1186/s12913-022-08596-w.

## Background

A prescription for medication has a central place in the current practice of health care reflecting the prominence of pharmacotherapy. It is not without complications, however, and the literature is filled with data about overuse, underuse, misuse, and side effects of drug use [1, 2]. The causes of these problems do not come solely from the drugs themselves, but also from how prescriptions are formulated [3] and executed [4]. There clearly can be more to a prescription than just the name and dose of a particular drug although the exact nature of this communication between health care workers has been the subject of contentions debates for decades [5–7]. Ultimately, however, this communication should benefit the patients as their safety is at stake [8, 9].

Including an indication or a ‘reason for use’ is, with a few exceptions, a missing element in the medication prescribing process [10–15]. Schiff et al. delineate five aspects that are important to guarantee the safety of medication use and delivery: (i) the right patient, (ii) the correct drug, (iii) the appropriate dose, (iv) the correct time, and (v) the appropriate dosage form. Information about the indication is in their view a sixth essential aspect to guarantee a safe course of medication use and delivery [11]. When the ‘reason for use’ must be included on prescriptions, prescribers have sometimes expressed concern about the greater workload, the confidentiality of the information, the liabilities connected to off-label prescribing, the difficulty of expressing this in an accessible manner in addition to concerns about clinical autonomy [11].

The current research on including ‘the reason for use’ was recently and comprehensively reviewed by Mercer et al. [16] using that specific phrasing and therefore, in this manuscript, the term ‘reason for use’ is utilized unless a direct reference to another study is made. Some data is available about the opinions of physicians and pharmacists regarding this possible extra aspect of the prescription [17–19]. Pharmacists are undoubtedly the long-time leading enthusiasts as they perceive this extra information a prerequisite for providing better pharmaceutical care. From their point of view, including reason for use could help identify and correct medication errors and could eliminate unnecessary time-consuming contacts with prescribers [20–23]. In contrast to pharmacists, the opinions of prescribers were more diverse and on average clearly less supportive. Concerns about privacy are expressed and there is some apprehension about the extra effort needed to include a ‘reason for use’ [19, 21]. These dispositions from the literature were also present in our survey in 2019 of pharmacists and physicians in Flanders [24]. In the US, a survey showed that the indication was present in 8% of the prescriptions [15]. Furthermore, in the Netherlands, the uptake of the mandatory inclusion of the indication for a select subgroup of medications was also low at 13% [25].

Patient opinions on the inclusion of a ‘reason for use’ have been much less studied [16], mostly through focus groups or through other qualitative research methodologies [13, 19, 21, 25]. Bearing this in mind, a study of the public opinion on these matters in Flanders, Belgium was initialed through an online survey. At the time of the study there were no regulations or software implementation features mandating of restricting the addition of extra information, such as a ‘reason for use’ to prescriptions but its inclusion in primary care is very rare. It should also be made clear that in Belgium prescriptions are routinely made available to insurance companies for auditing purposes.

## Methods

A Dutch questionnaire was prepared to investigate the opinion of the general public on including a ‘reason for use’ (the condition) on prescriptions for medication. Its design was an iterative process with the active participation of all authors. The different themes resulting from the qualitative studies in the literature [13, 16, 19, 21, 24, 25] guided the selection of topics in the questionnaire. Interim versions were piloted among a small number of people without a professional medical background. A final version with 37 questions was transformed into an online questionnaire with the QualtricsXM software [26]. An internet-based questionnaire, consisting mainly of 5-point Likert scale questions, labelled with ‘Strongly agree’, ‘Agree’ ‘Neutral’, ‘Disagree’,‘Strongly disagree’, was used to register the opinions of the participants. An English translation of the questions asked can be found in the in supplementary material. These were preceded by a few general demographic questions (see Table 1) and a question about whether the respondents were practicing physicians or pharmacists, or were in training to become one.

**Table 1:** Participant’s demographics

	N	% of Total
**Gender**		
Male	242	23.2
Female	801	76.8
**Age**		
16–20 year	34	3.3
21–30 year	222	21.3
31–40 year	110	10.6
41–50 year	158	15.2
51–60 year	234	22.4
61–70 year	204	19.6
71–80 year	68	6.5
81 year or older	13	1.3
**Highest degree**		
None	4	0.4
Primary education	21	2.0
Secondary school	290	27.8
Higher education (not university)	486	46.6
University	242	23.2

The study was approved by Ethical committee of the Antwerp University Hospital (Belgian registration number: B3002020000202), informed consent was obtained from all subjects for study participation. The survey was online for 1 month starting on the 3rd of November 2020.

The survey was distributed in a pragmatic way through social media channels, primarily Facebook, and through a number of websites including patient organizations (i.e. Flemish patient platform (www.vlaamspatientenplatform.be), Diabetes Liga (www.diabetes.be)), and insurance organizations (Christian health insurance fund (www.CM.be)), the website of Plus Magazine (www.plusmagazine.be), Seniorennet (www.serniorennet.be) and the Flemish network of pharmacists (www.VAN.be).

For the statistical analyses JMP pro 15 was used and a chi-square test was performed. A *p*-value of < 0.05 was considered significant. All reported *p*-values were those corrected for multiple comparisons which the Bonferroni-Holm method [27] using IBM SPSS statistics software [28]. Heatmap creation was performed in R using the package pheatmap [29] and illustrates the order and extent of agreement among participants asked to rank a list presented to them in a random order.

## Results

### Response to the online survey

In total 1433 participants started the online survey. Of these, 26 participants refused to give permission to process the data after reading the information sheet, one participant was under the age of 16, and 177 participants did not fully complete the survey; this resulted in 1229 valid responses. Among these responses, 144 originated from pharmacists or pharmacy students, and 42 from physicians or medical students. There was no attempt to preemptively exclude these two stakeholder groups from our survey. Instead, these two groups were excluded from the main data analysis and analyzed separately. The median time required to complete the questionnaire was 9 minutes and 9 seconds. The characteristics of the remaining 1043 respondents are shown in Table 1. Full results are given in the appendix together with the translated questionnaire.

### Opinion of the general public on the inclusion of a ‘reason for use’ on the prescription

**Fig. 1:**
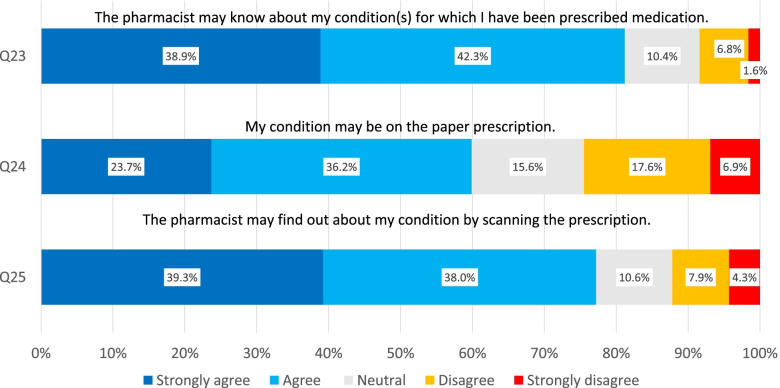
Opinions on including ‘reason for use’ on prescriptions. Responses (in %) on Question 23–25 of the questionnaire

On the question whether the pharmacist may know the medical condition(s) for which the prescription was made, a majority of 81.2% agreed or strongly agreed, (Fig. 1) and a smaller majority (59.9%) had no objection to this condition being present on the paper prescription. This number increased to 77.3% when a digital readout would be the only possibility to share the reason of use. This digitalization reflects an increase in favor of ‘strongly agree’ from 23.7 to 39.3%.

A reply to the open question was informative as to the reason for this increase in agreement to share the reason of use when using a digital readout: “If you send someone to the pharmacy on your behalf, it is not necessary that this person knows what condition(s) you have.”

On the general question if the pharmacist can give better advice upon knowing the disease affecting the patient a majority agreed or strongly agreed (80.7%). These answers were statistically very strongly correlated with those from the question whether the pharmacist may know the medical condition(s) for which the prescription was made (all *p*-values < 0.003).

In contrast, the willingness to have the condition labeled on the medicine package was smaller: 44.1% did not agree, 36.7% (strongly) agreed and 19.4% remained neutral. The open question pointed towards a possible rationale: “For many people, the medication is on the kitchen table or the cupboard. Not every visitor should be able to read ….” . Another participant stated that she did not want the daily confrontation with her condition being breast cancer. Advantages of the medication label were however also mentioned: “( …) I notice that my grandparents have no idea why or for what indication they take certain medicines. That actually means a loss of autonomy”. In general, 87% of the respondents agreed or strongly agreed with the statement that they personally knew the purpose of their prescribed medication (Q22).

### Relationship with a particular pharmacy

Questions 12 to 14 investigated whether the respondents usually went to the same pharmacy and delved into possible reasons. A large majority agreed or strongly agreed (93.9%) that they usually frequented the same pharmacy. Of all responders, 80.9% agreed or strongly agreed that they usually go to the same pharmacy because of location (Q14), and 73.1% usually go to the same pharmacy because of the trust they have built with the pharmacist (Q13). These responses were strongly correlated with responses to question 23: loyalty in pharmacy choice was strongly related to the acceptance of including of the ‘reason of use’ on prescriptions (all *p*-values < 0.003). Patients self-identifying as having a chronic condition were statistically more willing to share their ‘reason for use’ compared to non-chronic patients. In the opinion of the participants 54.6% agreed and 9.7% strongly agreed that a pharmacist can deduce the condition of the patient based on the prescribed medicines. There was a strong correspondence between having a chronic condition and the level of agreement that this information can be shared on the prescription (*p*-values < 0.005). The number of prescription medicines used served as a significant determinant of the inclusion of the ‘reason for use’ in the reasons (data not shown).

### Is the opinion of the public receptive to additional information?

**Fig. 2:**
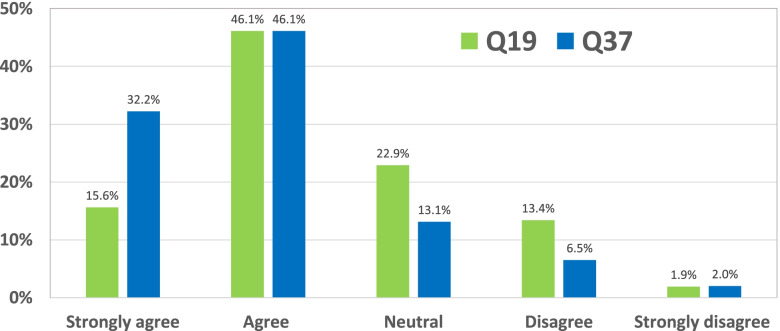
Impact of extra information on the opinions on including ‘reason for use’ on prescriptions. Responses (in %) on Q19 ‘With a condition on the prescription, the pharmacist would be better able to prevent medication errors.’ compared to same question Q37 after obtaining some information

As in previous research [30] we probed if additional information provided during the questionnaire altered the opinion of the respondents. Question 19, asking whether a pharmacist would be better able to prevent medication errors when a ‘reason for use’ was linked to the prescription, was therefore repeated at the end of the questionnaire (Q37) after a brief explanation on how different doses of methotrexate are used in different clinical situations (see questionnaire in the appendix). The results are shown in Fig. 2.

Comparison of the answers to the same question (Q19 and Q37) “With a condition on the prescription, the pharmacist would be better able to prevent medication errors.” as influenced by a brief note about disease dependency of the dose of methotrexate.

Although this extra information did not change the opinion of the 2% minority who was already strongly opposed to ‘reason for use’ mentioning, a marked shift towards more willingness was observed and the share of those strongly agreeing doubled and reached 32%. ‘Agree’ and ‘Strongly agree’ increased from 61.7 to 78.3%.

### Is the opinion of the public dependent on the specific condition?

**Fig. 3:**
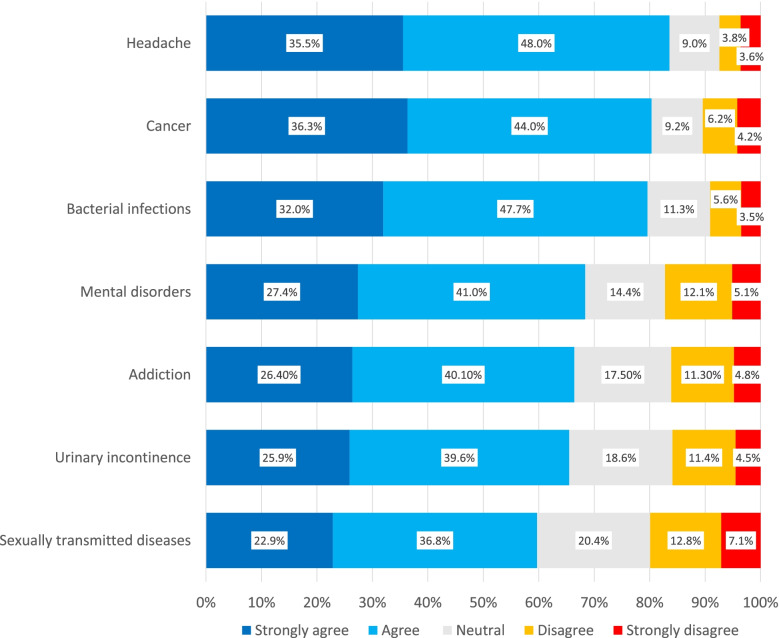
Impact of the type of condition on the opinion concerning inclusion of ‘reason for use’ on prescriptions. A representative subset of conditions queried in Q30 sorted from higher to lower acceptance

Whether different conditions have different sensitivities was investigated in the latter part of the questionnaire. In Fig. 3 the acceptance of mentioning the ‘reason for use’ for a subset of conditions are shown (full results in the appendix).

**Fig. 4:**
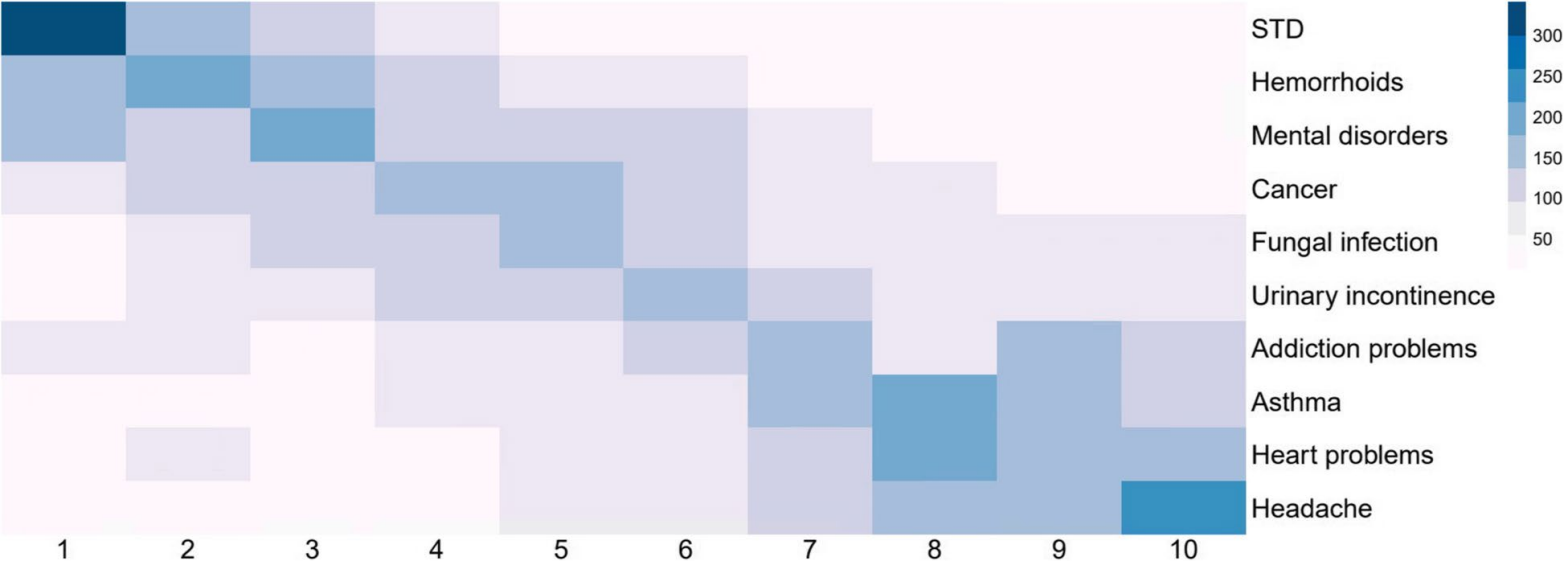
Ranking of conditions in order of concern on mentioning the condition on the prescription. Heatmap derived from the rankings given by respondents. Participants were asked to rank how problematic the above pathologies were in terms of the being included as part of the ‘reason for use’ on prescriptions. The most sensitive condition had to be placed at number 1 and the least sensitive at number 10 starting from a randomized list generated anew for each participant. The average ranked position determined the sequence in the figure. The darkness of the colour reflects the relative frequencies of each pathology in that position of the ranking. Consensus in assessing STD’s as most sensitive is thus illustrated in the dark upper left corner. In contrast, headaches, on the other side of the spectrum, are universally perceived as most acceptable. (STD Sexually transmitted diseases)

As can be expected, acceptability decreased with certain indications. However, a clear majority still agreed to share the reason of use on the prescription regardless of the indication. With the sensitive matter of STDs (Sexually transmitted diseases), a maximum of 19.8% disagreed. Headache caused the least opposition as 7.5% disagreed. Respondents were also asked to rank these conditions, from most to least concern. The results can be seen in Fig. 4 and confirm the answers from Q30.

Clearly there is broad consensus about the conditions and the hierarchy that cause the most concern; STDs followed by mental illness and hemorrhoids. On the other side of the spectrum, headaches clearly elicit the least concern next to a cluster of conditions consisting of heart disease and asthma.

### Opinions about the storage and transmission of the ‘reason for use’ information

In Table 2 responses to Q32 to Q35 on this topic were summarized.

**Table 2:** Participant’s responses to questions about the data-handling of the ‘reason for use’ in %

	Strongly agree	Agree	Neither agree nor disagree	Disagree	Strongly disagree
Q32: A pharmacist may, with my permission, pass on the information about my condition to other pharmacists and physicians.	14.8	45.5	13.4	16.8	9.6
Q33: A pharmacist may, without my permission, pass on the information about my condition to other pharmacists and physicians.	2.4	3.9	5.9	34.2	53.5
Q34: A pharmacist may save information about my condition in a database that can only be consulted by the same pharmacist (or a fellow pharmacist from the same pharmacy).	15.7	50.2	14	11.5	8.5
Q35: A pharmacist may save information about my condition in a database that can be consulted by various pharmacists from other pharmacies.	4.9	13.7	17	33.9	30.5

Overall, the responses to these questions reveal that participants like to control the ultimate use of this potentially sensitive information. A clear majority agrees to make this information available for the team of the single pharmacy of their choice (65.7%). However, a similar majority (64.4%) does not agree to share this information automatically among other pharmacies without their permission.

### Opinions of physicians and pharmacists

Physicians and pharmacist, and the respective students, were excluded from the main analyses because of the patient-oriented scope of this investigation. Although the number of respondents is smaller, these expressed opinions can still be informative. For example, in comparison with the data in Fig. 1, 90% of the pharmacists agreed with Q24 in contrast to physicians where only 36% agreed. An increase to respectively 97 and 60% was noted if the condition was only transmitted electronically. In contrast to the general public, these opinions were not significantly influenced by the additional information about methotrexate.

## Discussion

This questionnaire-based study was initiated to investigate the opinion of the general public concerning inclusion of ‘the reason for use’ on a medical prescription. Because of a lack of quantitative data on this topic and because of the constraints inherent to the COVID-19 situation, an internet-based questionnaire was used. Even before the COVID-19 crisis, this became the predominant survey methodology [31], although it can introduce a certain amount of bias [31–34] as those with limited digital skills or those not fluent in Dutch may not be reached [35]. This kind of bias is, to a certain extent, inherent in all questionnaire studies [36]. The COVID-19 situation will undoubtedly have further familiarized numerous people in Flanders to online surveys [37, 38]. In addition an online questionnaire may have reduced the risk of bias linked to the style and additional instructions provided by the in-person interviewer [34]. Independent from its online implementation, the convenience sampling used in our study is another limitation that could have led to underrepresentation of certain groups, limiting the generalizability of the findings. The survey was, by design, kept short in order to maximize fully completed responses. On average, less than 10 minutes were needed, well below the 13 minute limit as has suggested by Fan et al. [39].

Experts have clearly spelled out the advantages and disadvantages of including a ‘reason for use’, [11, 40] but a successful implementation is rare, particularly in primary care [41]. The range of opinions connecting barriers and disadvantages with resistance, hesitancy, or opposition to implementing these changes are well known from qualitative studies [13, 19, 21, 25] and guided the development of the questionnaire with the aim to inform this debate with objective, quantitative data.

The results point toward a large majority of our respondents which are open to the idea of more information transfer from the physician to the pharmacist concerning the condition that led to the prescribing. This opinion is positively related to being prescribed multiple drugs, as this presumably induces more frequent contact with the pharmacist. People who are already convinced that this increases the potential for the pharmacist to intervene on medication errors and those who realize that the pharmacist can already deduce the condition in a sizable number of cases, not surprisingly, also favor this inclusion. The link with a particular pharmacy/pharmacist is important on several levels: (i) people who have high loyalty to a particular pharmacy are more in favor of information transfer; (ii) people object to the further dissemination of this information beyond their habitual pharmacy. This is important considering the actual existence in Belgium of a shared personalized database of dispensed medicines quarriable in all pharmacies [42]. The relative reluctance to having a condition labeled on the medicine package or explicitly printed on the paper prescription, can be seen in the same context: people want this information to selectively reach the pharmacist and not the occasional third person who picks up the medication for them or a visitor spotting the medicine package in the cupboard. There is a certain correspondence to some of the objections expressed by physicians in the use of this information beyond that particular pharmacists/pharmacy. This data may, for example, reach insurance companies and trigger reimbursement issues and there is the obvious fear of legal liability in the context of off-label prescribing.

The questions concerning the mentioning of specific diseases had a predictable gradient of acceptability, but even for STDs a majority of the respondents did not object. This gradient in acceptability could certainly inform a potential practical implementation order and therefore increase patient acceptability, although a selective approach introduced in the Netherlands was not particularly successful [25].

Instead of trying to discourage the main stakeholders, physicians and pharmacists, from participating in the survey, their responses were analyzed separately. The smaller number precludes an in-depth analysis but, not surprisingly, the quantitative trends reflect what the literature [16] and our previous experiences [24] already conveyed us: near universal enthusiasm among pharmacists and a very divide opinion among physicians. Extra information about disease specific doses of methotrexate does not influence these opinions in stark contrast to the general public.

One main conclusion of this study is that information about the advantages on the information transfer of the ‘reason for use’ to the pharmacists must be communicated with the patients to increase acceptability of a potential policy initiative. With the support of the majority of patients other implications for practice are the possible increase in patient safety by preventing dosing errors and improved pharmaceutical care through better patient counselling about medication with multiple uses.

Overall, this data further informs the debate on the information transfer from the prescribing physician to the pharmacists. It is a concept that is supported by a big majority of patients, but this support is conditional on several qualifications. The data also indicates that it is not productive to simplify everything to a binary debate: a simple yes or no to tightly couple the prescription of a particular drug with a particular ‘reason for use’. In the context of increasing digitalization of the prescription process this tight linking may increase resistance among patients because of the fear of further use. The digitalization without tight coupling and information transfer solely to the pharmacy chosen by the patient may on the other hand increase the acceptability and alleviate other privacy objections such as the pickup of medication by a third party. Additional research should extend these findings to other regions and further explore the implementation issues.

## Conclusion

The data presented document strong support by patients for the information transfer to their regular pharmacy, in a secure and privacy conscious way, of the ‘reason for use’ linked to a particular prescription. The data also show that patient support for this change in practice can be broadened by providing information about patient-safety benefits and by taking into account the sensitivities around certain conditions.

## Supplementary Information

**Additional file 1.**

## Data Availability

The dataset generated and analyzed during the current study is available in the [figshare] repository with the identifier [DOI 10.6084/m9.figshare.19210278].
